# Clinical Picture in Hematology

**DOI:** 10.4274/tjh.2014.0359

**Published:** 2015-02-15

**Authors:** Şinasi Özsoylu

**Affiliations:** 1 Retired Professor of Pediatrics, Hematology, and Hepatology, Honorary Fellow of American Academy of Pediatrics, Honorary Member of American Pediatric Society

**Keywords:** Microcytosis, Zinc deficiency

## TO THE EDITOR

I am writing this letter concerning the “Clinical Picture in Hematology” entitled “Isolated Zinc Deficiency Causing Severe Microcytosis and Sideroblastic Anemia” by Shweta et al. in a recent issue of this journal [1].

Although we have seen several cases of zinc and iron deficiency with geophagia, hepatosplenomegaly, growth retardation, and hypogonadism (Tayanç-Reimann-Prasad syndrome) [2] and zinc deficiency with acrodermatitis enteropathica and immunodeficiency, the authors’ case looked completely different than these other syndromes, with high ferritin and bone marrow ring sideroblasts (low zinc level was most likely an associated finding).

Although the authors’ working diagnosis of “myelodysplastic syndrome (MDS, refractory anemia with ring sideroblasts)” seems to be the best choice, I would like to question probable pyridoxine administration in mineral supplements that contained copper and zinc “amongst” others.

Among other possibilities, hepcidin, heme oxygenase heterozygosity [3], chronic liver disorders [4], and the presence of α-thalassemia (hereditary or acquired because of the proband’s origin of birth and erythrocyte morphology) should be investigated by hemoglobin electrophoresis at pH 6.4, and H inclusion bodies should be determined for a better explanation in this very unusual case, which might shed light on iron and zinc metabolism, sideroblastic anemia, and iron homeostasis at large, I believe.
